# Management of Chronic Portal Vein Thrombosis in a Cirrhotic Patient With Pancytopenia and Grade II Esophageal Varices

**DOI:** 10.7759/cureus.21150

**Published:** 2022-01-12

**Authors:** Ahmed Ali Aziz, Daoyu Yang, Muhammad Naeem, Donald Christmas

**Affiliations:** 1 Internal Medicine, Jersey Shore University Medical Center/Saint Francis Medical Center, Trenton, USA

**Keywords:** hepatitis c virus infection, acute portal vein thrombosis, esophageal varices, “pancytopenia”, systemic anticoagulation, anticoagulation, cavernous transformation of the portal vein, liver cirrhosis, liver fibrosis, chronic portal vein thrombosis

## Abstract

Chronic portal vein thrombosis (PVT) is a major vascular complication of liver cirrhosis. Patients may be asymptomatic and chronic PVT might be detected incidentally on imaging. PVT is associated with worsening liver disease, poorer clinical outcomes, and might proceed to life-threatening intestinal ischemia. Management of chronic PVT with anticoagulation has been shown to be successful in promoting recanalization and reducing thrombus extension in patients with cirrhosis. However, optimal anticoagulation for PVT in cirrhosis has not yet been addressed in any large-scale trial, and the decision to anti-coagulate varies on a case by case presentation. We report the case of a 62-year-old male patient with a history of liver cirrhosis, pancytopenia, and grade II esophageal varices presenting with abdominal pain who was incidentally found to have chronic thrombosis of the portal vein on imaging and was managed appropriately with a good outcome.

## Introduction

Chronic portal vein thrombosis (PVT) is one of the major vascular complications of advanced liver cirrhosis. Patients may be asymptomatic and chronic PVT might be detected incidentally on imaging [1]. Reduced portal blood flow and increased resistance to portal blood flow are the main etiologies for the formation of PVT in cirrhosis [2]. Chronic PVT is differentiated from acute PVT by the presence of venous collaterals that bypass the occluded segment called "cavernous transformation" or "cavernoma of portal vein" detected on imaging [3,4]. Complications of chronic PVT include variceal hemorrhage, intestinal ischemia, and portal biliopathy [5]. Ultrasound and CT imaging can both be used for the diagnosis of PVT [1]. Management of PVT in cirrhosis involves anticoagulation. However, optimal anticoagulation for PVT in cirrhosis has not yet been addressed in any consensus and the decision to anti-coagulate varies case by case presentation [1]. In this case report, we present a 62-year-old male patient with a history of liver cirrhosis, pancytopenia, and grade II esophageal varices who presented with abdominal pain and was incidentally found to have PVT on imaging. He was timely diagnosed and managed appropriately, with a good outcome.

## Case presentation

**Figure 1:**
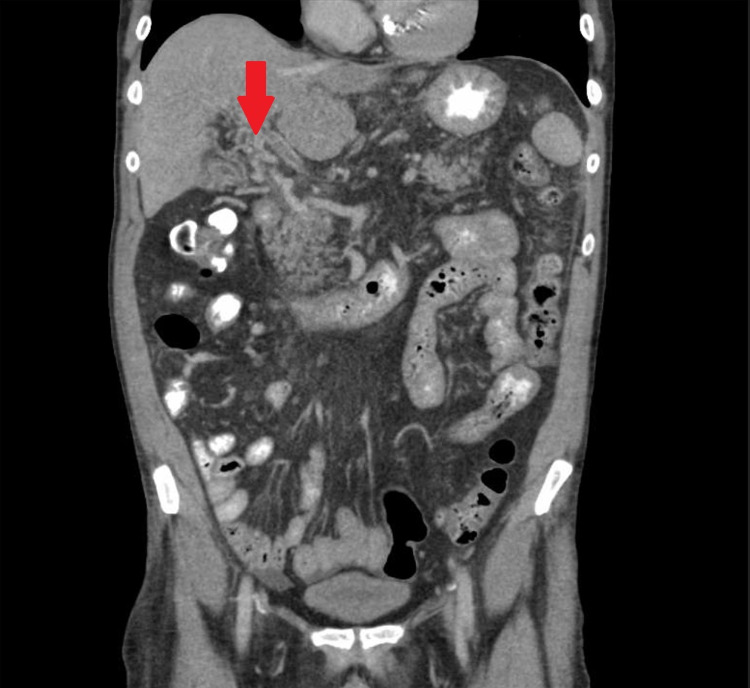
CT scan of the abdomen with IV contrast (coronal view). Arrowhead demonstrates the cavernous transformation of the portal vein and thrombus within the portal vein.

**Figure 2:**
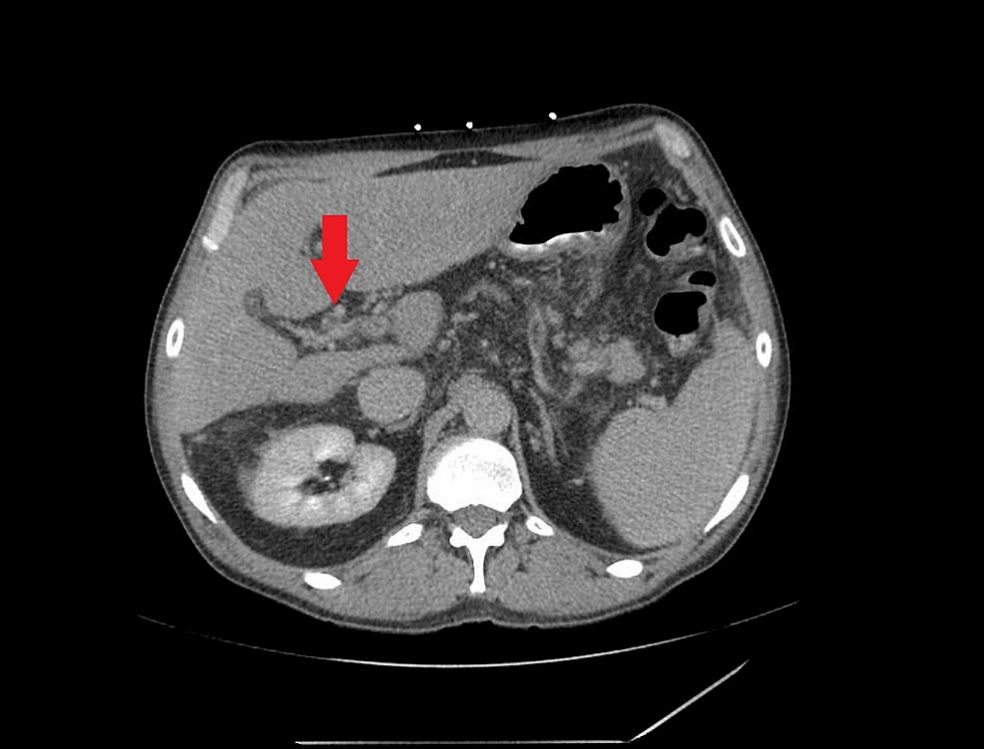
CT scan of the abdomen with IV contrast. Arrowhead demonstrates the cavernous transformation of the portal vein.

A 62-year-old male with a past medical history of liver cirrhosis, untreated hepatitis C virus infection, and grade II esophageal varices was admitted for a chief complaint of diffuse cramping abdominal pain and non-bloody diarrhea ongoing for four days after eating fast food. On examination, his blood pressure was 124/62 mm Hg, pulse was 67 beats per minute, respiratory rate was 18 breaths per minute, the temperature was 98.5°F, and oxygen saturation was 100% on room air. The abdomen was distended, non-tender to palpation with dullness to percussion at the bilateral flanks, and normal bowel sounds. Initial blood work revealed hemoglobin 7.3 g/dL (normal: 14-17 g/dL), hematocrit 21.5% (normal: 41%-51%), WBC 2.3 K/uL (absolute neutrophil count 1.0 K/uL) (normal: 3.3-8.7 K/uL), platelets 78 K/uL (normal: 147-347 K/uL), serum aspartate aminotransferase (AST) 56 U/L (normal: 0-35 U/L), alanine aminotransferase (ALT) 45 U/L (normal: 0-35 U/L), alkaline phosphatase 77 U/L (normal: 36-92 U/L), total bilirubin 0.8 mg/dL (normal: 0.3-1.0 mg/dL), albumin 2.5 mg/dL (normal: 3.5-5.0 mg/dL), and international normalized ratio (INR) 1.2. A CT scan of the abdomen and pelvis with IV contrast showed PVT with cavernous transformation (Figures 1, 2). Given the presence of cavernous transformation of the portal vein on CT scan, the thrombus was likely chronic, and abdominal pain was likely secondary to foodborne illness or viral gastroenteritis that resolved on the next day of admission. Management of chronic PVT requires anticoagulation. However, prior to starting anticoagulation, patients require a risk assessment for bleeding. This risk assessment especially includes evaluation for any upper GI varices. Our patient had an outpatient endoscopy three months prior to admission that revealed grade II esophageal varices (classified as high risk for bleeding). Since he was at high risk for bleeding due to esophageal varices and had pancytopenia; a multidisciplinary approach was adopted involving gastroenterology and hematology-oncology to discuss the need for anticoagulation for the patient. The decision was made to monitor the patient without anticoagulation as the risks of anticoagulation greatly outweighed the benefits. The patient was discharged without anticoagulation and was on telemedicine follow-up two weeks and two months later; he was doing well.

## Discussion

PVT is one of the major vascular complications of advanced liver cirrhosis and is often found incidentally in asymptomatic patients [1], as in our patient who was symptomatic due to a foodborne illness and was incidentally found to have PVT on CT scan of the abdomen and pelvis. Increased extra-hepatic portal resistance and reduced portal blood flow are the main factors responsible for the formation of PVT in cirrhosis [2,6,7]. The prevalence of PVT in cirrhotic populations is 0.6% to 26% [8].

Acute PVT involves the formation of a new (either partially or completely) occlusive thrombus in the portal vein. In cirrhosis, however, the onset and progression of PVT is a slower process that allows the development of venous collaterals that bypass the occluded segment forming a cavernoma called the cavernous transformation of the portal vein. This usually takes three to five weeks to form [1]. Acute PVT can be differentiated from chronic PVT by the absence or presence of these cavernoma on imaging [3,4]. Our patient had the cavernous transformation of a portal vein on imaging and hence likely had chronic PVT. Complications of chronic PVT include variceal hemorrhage, intestinal ischemia, and portal biliopathy [4].

As mentioned above, management of chronic PVT requires anticoagulation to reduce chronic PVT complications. However, optimal management of chronic PVT with anticoagulation has not yet been well established in any large-scale research trial and the decision varies case by case [1]. For the management of patients with chronic PVT (either partial or complete), a prospective cohort study showed that treatment with low molecular heparin (nadroparin) resulted in a 60% incidence of complete recanalization versus 5% recanalization in the control group (without anticoagulation), with 71% progression of thrombosis in the control group as compared to the treatment group [9]. Anticoagulation can also be used for the primary prevention of PVT in cirrhosis [10]. A randomized, controlled study of enoxaparin showed there were no PVTs in the treatment group (patients with cirrhosis on anticoagulation) at the end of follow-up at two years, compared with the 27.7% rate of PVTs in the control arm (patients with cirrhosis off anticoagulation) [10].

Our patient had chronic PVT. He would have benefited from anticoagulation enabling recanalization and preventing the extension of thrombosis. However, he was at high risk of bleeding on anticoagulation as he had grade II esophageal varices and pancytopenia. Since anticoagulation for chronic PVT in cirrhosis has not yet been agreed upon in any consensus, after a multidisciplinary approach involving hematology-oncology and gastroenterology we decided to monitor the patient without anticoagulation and he did well.

## Conclusions

Chronic PVT develops in liver cirrhosis secondary to increased resistance to blood flow in the portal circulation and is differentiated from acute PVT by the presence of cavernous transformation of the portal vein on imaging. Anticoagulation for the primary prevention of PVT in cirrhosis has been shown to effectively prevent the development of PVT. Anticoagulation for chronic PVT in cirrhosis has been shown to promote recanalization in 60% of patients, hence preventing PVT complications like intestinal ischemia and variceal hemorrhage. However, patients need to undergo a bleeding risk assessment prior to initiating anticoagulation for chronic PVT, including upper GI endoscopy, to rule out dilated varices at risk for bleeding. There are no exact guidelines for anticoagulation in chronic PVT and the decision varies case by case. Patients that are at high risk for bleeding or bleeding complications should be monitored without anticoagulation if the harms of anticoagulation exceed the benefits.
